# Rice ragged stunt virus-induced apoptosis affects virus transmission from its insect vector, the brown planthopper to the rice plant

**DOI:** 10.1038/srep11413

**Published:** 2015-06-15

**Authors:** Hai-Jian Huang, Yan-Yuan Bao, Shu-Hua Lao, Xiao-Hui Huang, Yi-Zhou Ye, Jian-Xiang Wu, Hai-Jun Xu, Xue-Ping Zhou, Chuan-Xi Zhang

**Affiliations:** 1 State Key Laboratory of Rice Biology and Ministry of Agriculture Key Laboratory of Agricultural Entomology, Institute of Insect Sciences, Zhejiang University, Hangzhou 310058, China; 2 Institute of Biotechnology, Zhejiang University.

## Abstract

Most plant viruses that seriously damage agricultural crops are transmitted by insects. However, the mechanisms enabling virus transmission by insect vectors are poorly understood. The brown planthopper (*Nilaparvata lugens*) is one of the most serious rice pests, causing extensive damage to rice plants by sucking the phloem sap and transmitting viruses, including *Rice ragged stunt virus* (RRSV). In this study, we investigated the mechanisms of RRSV transmission from its insect vector to the rice plant *in vivo* using the terminal deoxynucleotidyl transferase dUTP nick-end labeling assay and RNA interference technology. RRSV induced apoptosis in the salivary gland cells of its insect vector, *N. lugens*. The RRSV-induced apoptosis was regulated through a caspase-dependent manner, and inhibition of the expression of *N. lugens caspase-1* genes significantly interfered with virus transmission. Our findings establish a link between virus-associated apoptosis and virus transmission from the insect vector to the host plant.

## Introduction

Insect pests and plant viruses that are transmitted by insect vectors are major biological threats to crop production. The interactions between insect vectors and plant viruses have received extensive attention worldwide because of their importance in the agricultural sector. The most economically devastating insect vectors are restricted to a few Hemiptera and Thysanoptera taxa, such as planthoppers, aphids, whiteflies and thrips. The long-distance migrating brown planthopper, *Nilaparvata lugens* Stål (Hemiptera: Delphacidae), is considered one of the most destructive pests of rice throughout Asia, causing severe damage to rice plants by sucking the rice phloem sap and transmitting plant viruses, including *rice grassy stunt virus* (RGSV) and *rice ragged stunt virus* (RRSV)^1^,^2^.

RRSV is a member of the genus *Oryzavirus* in the family *Reoviridae* and causes rice ragged stunt disease. The disease was first discovered in 1976–1977 in Indonesia and the Philippines^3^, and then became prevalent in most rice-growing regions in South East Asia and southern China^4^,^5^. Since its discovery, RRSV has become one of the most important rice pathogens in these regions. RRSV is known to be horizontally transmitted by the brown planthopper in a persistent propagative manner but is not vertically transmitted via eggs or rice seeds^6^. *Nilaparvata lugens* acquires the virus by sucking the sap of infected rice plants. The virus first enters the epithelial cells of the midgut, where it proceeds to the visceral muscles surrounding the midgut, then disperses throughout the visceral muscles of the midgut and hindgut, and finally reaches the salivary glands^2^. During subsequent sap feeding, the virus within the saliva is transmitted to the host rice plant^7^. Despite our understanding of the invasion route within the insect body, the mechanisms underlying the transmission of this virus from its insect vector to the rice plant remain largely unknown.

The salivary gland is vital to the biological success of insects via its principal secretory function, e.g., arbovirus transmission in mosquito depends on the function of salivary gland during blood feeding^8^. We are interested in investigating the responses of the salivary gland to RRSV infection because this organ provides the essential link for understanding virus transmission from the insect vector to the rice plant. There are several intriguing observations indicating that arboviruses induce apoptosis in the midgut and salivary glands of mosquitoes^8^,^9^. The apoptosis associated with arbovirus infections can affect vector competence in mosquitoes^10^. In insect tissues, cytopathologic changes such as virus-activated apoptosis could have potential effects on virus dissemination or release. However, until now there has been no solid evidence showing a causal relationship between virus-induced apoptosis and virus transmission.

The molecular pathways that regulate apoptosis in insects have been most clearly studied in *Drosophila melanogaster* and *Aedes aegypti* by far^11^,^12^,^13^,^14^. The core components in the apoptosis pathway are initiator and effector caspases, which are a family of cysteine proteases that are ubiquitously expressed as inactive zymogens^15^,^16^. Apoptotic signals trigger initiator caspases, which function to activate downstream effector caspases that lack long prodomains and the ability to self-activate, and lead to programmed cell death^14^,^17^. We identified five *caspase* homolog genes through searching the *N. lugens* genome and transcriptome datasets^18^,^19^,^20^,^21^,^22^. Three genes are highly homologous to each other and share significant sequence similarities with insect *caspase-1* genes^11^,^23^,^24^,^25^,^26^,^27^. Caspase-1, the first insect caspase to be discovered, was identified in *Spodoptera frugiperda*^28^. Insect caspase-1 is most closely related to the mammalian apoptotic effectors caspase-3 and caspase-7^29^. We also identified two long prodomain-carrying *caspase* homolog genes in *N. lugens*. One shares similar characteristics with insect *caspase-Nc* genes that feature caspase activation and recruitment domains (CARDs). The other homolog has significant sequence identities with *caspase-8* genes of several insect species, which contain the long prodomain regions with no homology to known characterized motifs. In this study, we focused on these *caspases* to determine their association with RRSV-induced apoptosis in *N. lugens* salivary glands. The silencing of *caspase-1* or *caspase-Nc* expression inhibited RRSV-induced apoptosis in the salivary glands. However, the silencing of *caspase-8* expression did not inhibit the occurrence of apoptosis in the RRSV-infected salivary glands. Our findings revealed that the interference of *caspase-1* expression significantly reduced RRSV transmission from *N. lugens* to the rice plant. This study provides new insights, in that virus transmission was accompanied by caspase-dependent apoptosis in the salivary gland, which addresses a so far unknown step of virus transmission in a monophagous sap-sucking arthropod herbivore and is important for better understanding of insect vector–virus–plant host interactions.

## Results

### RRSV proliferation in the salivary glands of *N. lugens*

**Figure 1:**
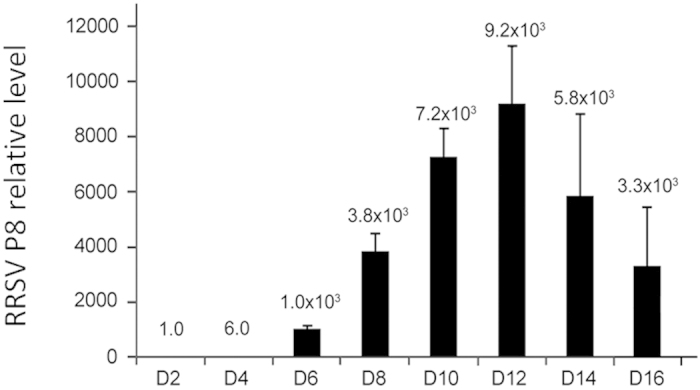
Relative quantification analysis of RRSV *P8* transcript levels in the salivary glands of *N*. *lugens*. Total RNA was extracted from salivary glands at the indicated times following RRSV infection and subjected to quantitative real-time PCR analysis using a pair of primers to specifically amplify the RRSV major capsid protein gene, *P8*. The relative *P8* transcript levels were calculated using *N. lugens 18S rRNA* as an internal control. A mixture of 100 salivary glands was used as one biological sample due to the very low quantity of salivary glands. Samples from each time point were tested in three biological replicates, and the mean value used to analyze the relative transcript levels.

**Figure 2:**
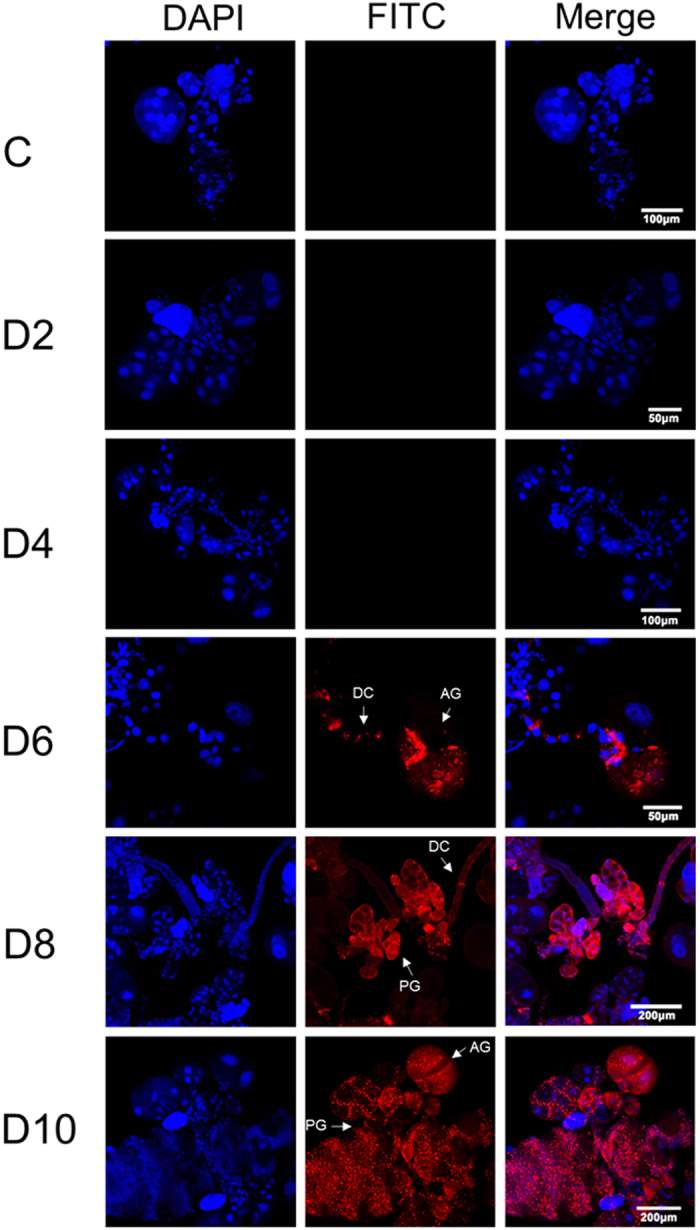
Confirmation of RRSV proliferation by immunofluorescence staining analysis. The salivary glands were dissected from RRSV-infected nymphs at the indicated times, and RRSV virions were immunolabeled with virus–FITC. Red (FITC) and blue (DAPI) fluorescence corresponding to the RRSV virions and the nuclei in the salivary gland cells, respectively. PG, principal glandular lobules; AG, accessory glands; DC, duct cells.

**Figure 3:**
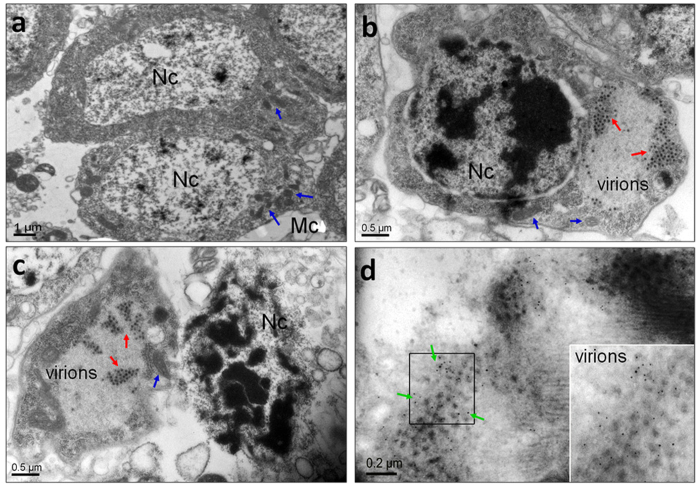
Identification of the location of the RRSV virions in the salivary gland cells at 8 d p.i. using transmission electron microscopy and immunoelectron microscopy. (**a**) The salivary glands of uninfected *N. lugens* nymphs. (**b** and **c**) The electron micrographs show RRSV virions distributed in the cytoplasm of the salivary gland cells. (**d**) Immunoelectron micrograph shows the immunogold labeling of RRSV in the cytoplasm of the salivary gland cells. The red arrows indicate the RRSV virions. The blue arrows show the mitochondria. The green arrows indicate 5-nm gold-conjugated goat-anti-mouse IgG against the RRSV major capsid protein P8 antigens. The inset shows the enlargement of the boxed areas. Nc, nucleus; Mc, mitochondria.

To understand the characteristics of RRSV invasion, we monitored the RRSV proliferation in the salivary glands after infection of the host, *N. lugens*. Quantitative real-time PCR analysis showed that specific amplification was achieved using one pair of primers against the RRSV major capsid protein *P8* gene, for which no nonspecific amplification or primer-dimer artifacts were observed. The relative transcript levels of the *P8* gene were barely detectable during 2–4 d p.i. (Fig. 1). As the infection progressed, viral proliferation increased rapidly, with a relative transcript level of 1.0 × 10^3^ at 6 d p.i. and a peak of 9.2 × 10^3^ at 12 d p.i., followed by a decrease to 3.3 × 10^3^ at 16 d p.i. These results reveal that the maximum accumulation of the virions in the salivary glands was at approximately 12 d p.i. The RRSV proliferation process was clearly visualized using immunofluorescence staining (Fig. 2). The virions were captured in the cytoplasmic region in a portion of the cells of the salivary gland tissues at 6 d p.i. and were then observed in more than half of the cells in these tissues at 8 d p.i. The rapid proliferation of the virus resulted in its occupying all salivary gland cells at 10 d p.i. The virions were not detectable during 2–4 d p.i. Further evidence of RRSV invasion was obtained by examining virus-infected salivary glands using TEM at 8 d p.i. (Fig. 3). RRSV has an icosahedral capsid of approximately 65–70 nm in diameter, which includes electron-dense cores of approximately 45–50 nm in diameter that are surrounded by outer shells of approximately 10 nm in width^2^,^30^,^31^. TEM scanning showed that virions of approximately the same size were distributed in the cytoplasm of the RRSV-infected salivary gland cells in clusters (Fig. 3b,c). Such virions were not detectable in uninfected salivary glands (Fig. 3a). Some virion-containing salivary gland cells show structural changes. Highly condensed chromatin in large compact granular masses was observed in the nuclear regions (Fig. 3b,c). In these cells, the nuclear membrane disappeared and/or invagination of the cell membrane formed. The structural changes indicate that these salivary gland cells were undergoing apoptosis. In contrast, uninfected salivary gland cells displayed a large nucleus with limited condensation of the chromatin localized around the nuclear membrane (Fig. 3a). To confirm whether the virions observed by TEM were RRSVs, we used IEM to localize the viruses in the salivary gland cells. There was 5-nm immunogold labeling against a RRSV P8 antigen detected in the cytoplasm of the salivary gland cells (Fig. 3d), consistent with the TEM results. These results clearly demonstrate the presence of RRSV in the cytoplasm of *N. lugens* salivary gland cells after virus infection. More importantly, TEM showed that RRSV infection induced apoptosis-related morphological changes in *N. lugens* salivary gland cells.

### RRSV induces apoptosis in salivary glands

**Figure 4:**
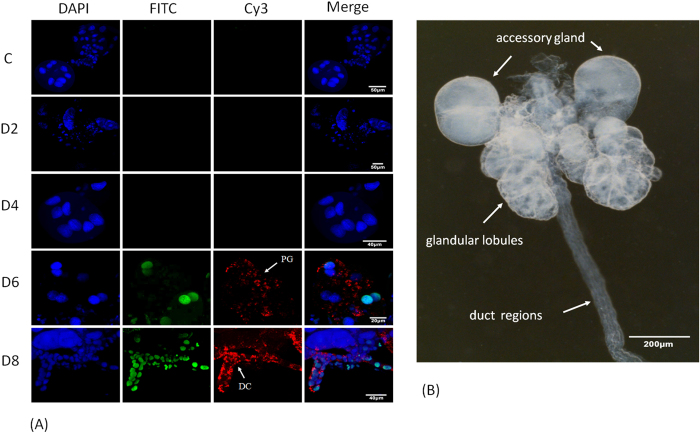
RRSV-induced apoptosis in the salivary glands of *N. lugens*. (**A**) The salivary glands were dissected from RRSV-infected *N. lugens* nymphs at the indicated times. Uninfected individuals were used as controls. The salivary glands were analyzed using a TUNEL assay to detect the apoptotic cells. Greenish-yellow fluorescence signals (FITC) indicate the nuclear regions of the principal glandular lobule cells and the duct epithelium cells undergoing apoptosis at 6 and 8 d p.i., respectively. Red fluorescence signals indicate the immunolabeled RRSV virions with virus-Cy3. Blue fluorescence (DAPI) shows the nuclei of the salivary gland cells. (**B**) Structure of the *N. lugens* salivary gland. The salivary glands were dissected and observed using a Leica S8AP0 stereomicroscope.

To investigate RRSV-associated apoptosis in the salivary gland, we examined virus-infected and uninfected tissues from 2–8 d p.i. using the TUNEL assay (Fig. 4A). Apoptosis was not detectable in salivary gland cells of uninfected individuals. Apoptosis was also not observed in salivary glands at 2 or 4 d p.i., possibly because RRSV had not yet spread to the salivary gland cells during this infection stage. However, apoptotic cells displaying significant greenish-yellow fluorescence signals, as visualized in the nuclear regions using a FITC filter, were found in clusters at 6 or 8 d p.i. Apoptosis occurred in the RRSV-infected cells that were indicated by red fluorescence when using the viral antibody conjugated to Cy3 to detect the virus. The *N. lugens* salivary glands are paired, sac-like, glandular lobules that anteriorly connect to a common duct that opens into the pharynx (Fig. 4B), and they consist of the principal glandular lobules, accessory gland and duct regions. We dissected the salivary glands from five pools of 200 RRSV-infected *N. lugens* nymphs at each time point. In the viruliferous insects, a portion of the salivary gland cells that included the principal gland cells and the duct epithelium cells were undergoing apoptosis at 6 and 8 d p.i. (Fig. 4A), while the accessory gland cells were not undergoing apoptosis. These results suggest that RRSV infection induced apoptosis in the salivary glands, but this was limited to a portion of the principal gland cells and the duct epithelium cells.

### Possible apoptosis-activating factors in RRSV-infected salivary glands

**Figure 5:**
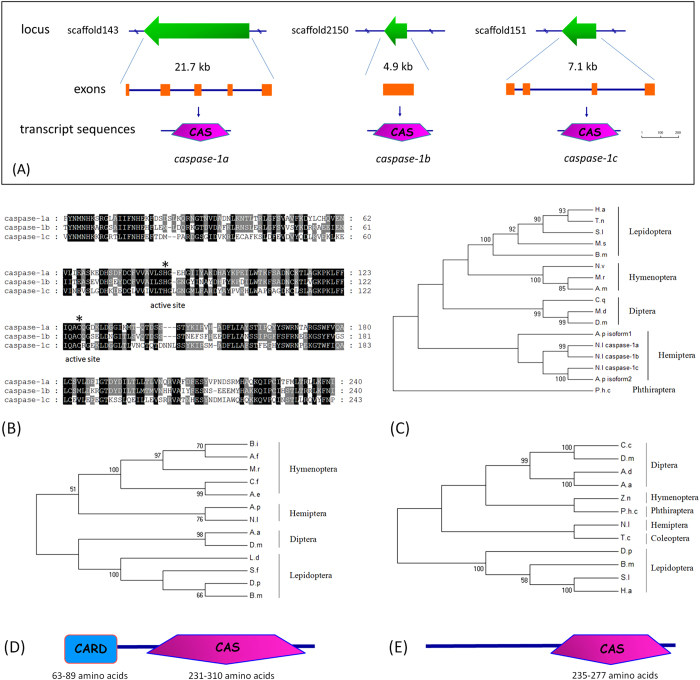
Identification of *N. lugens caspase genes*. (**A**) The gene organization of *N. lugens caspase* on scaffolds. The green arrows in the upper panel indicate the gene sizes and transcription orientations on the scaffolds. The transcript sequences of the *caspase* genes were matched to *N. lugens* genomic sequences to identify the exons and introns using the online tool Spidey (http://www.ncbi.nlm.nih.gov/spidey/spideyweb.cgi). The exons are indicated by orange boxes. The schematic representation of the deduced protein structures is shown under the gene structures. The caspase domains are indicated by pink pentagons. The size bar indicates the amino acid residues of the deduced proteins. (**B**) Sequence alignment of caspase domains of the deduced *N. lugens* caspase-1 proteins. The ClustalX program was used for alignments. The GenBank accession numbers for the sequences are as follows: *N. lugens caspase-1a* (KF956388), *caspase-1b* (KF956389), and *caspase-1c* (KF956390). The active-site histidine and cysteine residues required for caspase activity are marked by asterisks. The amino acids shaded in black and gray indicate the conserved and type-conserved residues, respectively. (**C**) Phylogenetic analysis of insect *caspase-1* genes. The phylogenetic tree was constructed by Maximum Likelihood using the program Mega 5.05 (http://www.megasoftware.net/). The Jones–Taylor–Thornton (JTT) for amino acid substitution model was used, while a test of phylogeny was carried out using the bootstrap method with 1000 replications; bootstrap values >50% are shown on each node of the tree. (**D**) Phylogenetic analysis and the predicted domains of the insect *Nc-like* genes. (**E**) Phylogenetic analysis and the predicted domains of the insect *caspase-8* genes. The CARD and caspase domains are indicated by blue box and pink pentagon, respectively. H.a, *Helicoverpa armigera*; T.n, *Trichoplusia ni*; S.l, *Spodoptera litura*; M.s, *Manduca sexta*; B.m, *Bombyx mori*; N.v, *Nasonia vitripennis*; M.r, *Megachile rotundata*; A.m, *Apis mellifera*; C.q, *Culex quinquefasciatus*; M.d, *Musca domestica*; D.m, *Drosophila melanogaster*; P.h.c, *Pediculus humanus corporis*; A.p, *Acyrthosiphon pisum*; N.l, *N. lugens*. A.a, *Aedes aegypti*; C.f, *Camponotus floridanus*; B.i, *Bombus impatiens*; A.e, *Acromyrmex echinatior*; A.f, *Apis florea*; D.p, *Danaus plexippus*; L.d, *Lymantria dispar*; S.f, *Spodoptera frugiperda*. T.c, *Tribolium castaneum*; Z.n, *Zootermopsis nevadensis*; C.c, *Ceratitis capitata*; A.d, *Anopheles darling*.

Caspases are central components of the machinery responsible for apoptosis in animal cells. However, significantly less is known about insect caspases other than those described in *Drosophila*. To understand the regulation mechanism involved in RRSV-induced apoptosis, we focused on *caspase* genes to investigate their roles in RRSV-associated apoptosis in *N. lugens* salivary glands. We identified five *caspase* homologs, as shown in a supplementary file 2, by searching the *N. lugens* genome and transcriptome sequences obtained from *N. lugens* salivary gland, fat body, gut, ovary, testis and integument tissues in our recent studies. Three genes were highly homologous to each other and shared significant sequence similarities with insect *caspase-1*s, the effector death proteases that are thought to execute apoptosis in insects. Gene structure analysis revealed that these *caspase-1* genes were located at different scaffolds with single or multiple (4–5) exons (Fig. 5A). Their predicted protein products (288, 271 and 258 amino acid residues, individually) possessed characteristic caspase domains (Pfam domain PF00656) that nearly spanned the entire coding regions of the putative proteases (Fig. 5B). We designated these genes as *Nlcaspase-1a*, *Nlcaspase-1b* and *Nlcaspase-1c*. Phylogenetic analysis showed that the *caspase-1* genes of Lepidopteran, Hymenopteran and Dipteran insects formed three major clusters (Fig. 5C). The homologous genes from two Hemiptera insect species, *N. lugens* and *Acyrthosiphon pisum*, formed an independent cluster and were closely related to each other, suggesting that they had the closest phylogenetic relationship among the compared insect species. Two other *N. lugens caspase* homologs encoded 686 and 604 amino acid residues containing the characteristic caspase domains and showed significant sequence similarities with insect *Nedd2-like caspases* (*Nc*) and *caspases-8*, respectively. We designated these two genes as *Nlcaspase-Nc* and *Nlcaspase-8*. *Nlcaspase-Nc* included a long amino-terminal CARD (Pfam domain PF00619), a specific protein–protein interaction motif, which is commonly considered to be the primary apoptotic initiator in the cell death pathway. A comparison of insect Nc-like proteins revealed that they are composed of a CARD sequence with 63–89 amino acid residues at the amino-terminus and a caspase sequence of 231–310 amino acid residues at the carboxyl terminus (Fig. 5D). The phylogenetic tree showed that Lepidopteran and Dipteran *Nc*-like genes formed a cluster, while the *N. lugens* and *A. pisum Nc*-like genes were in another cluster and were closely related to the homologous genes of Hymenopteran insects. *Nlcaspase-8* shared high similarities with insect *caspase-8/dredd* genes. Their deduced amino acid sequences contained a caspase domain with 235–277 amino acid residues and a long prodomain but lacking protein–protein interaction motifs that mark them as initiator caspases, unlike mammalian *caspase-8* with the death effector domains (DED) (Fig. 5E). *Nlcaspase-8* is phylogenetically most closely related to *Tribolium castaneum caspase-8* among the compared insect species (Fig. 5E).

**Figure 6:**
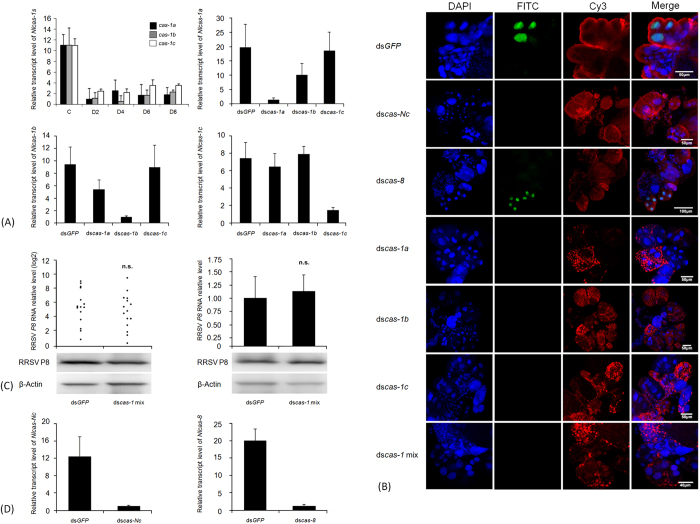
Analysis of the apoptosis-activating factors in RRSV-infected salivary glands. (**A**) The silencing of *Nlcaspase-1* gene expression in *N. lugens*. Second-instar nymphs were microinjected with the ds*caspase-1* mixture or each ds*caspase-1* individually. The relative transcript levels of each *Nlcaspase-1* gene were analyzed as described in Fig. 1. The expression silencing of the three *Nlcaspase-1* genes by microinjection with the ds*caspase-1* mixture is shown in the upper left panel. Black, grey and white boxes indicate the transcript levels of *Nlcaspase-1a*, *-1b* and *-1c* genes at the indicated time points after RNAi. The transcript levels of *Nlcaspase-1a*, -*1b* or -*1c* genes in ds*GFP*- and each ds*caspase-1*-treated nymphs are shown in the upper right, the lower left and right panels, respectively. (**B**) The RNAi effect on RRSV-induced apoptosis in *N. lugens* salivary glands. The nymphs were microinjected with the dsRNAs specific to the target genes and infected with RRSV. The salivary glands were dissected from the insects at 8 d p.i. The tissues were stained using a TUNEL assay to detect the apoptotic signals (green) and counterstained with DAPI to show the nuclei (blue) of the salivary gland cells. RRSV virions (red) were immunolabeled with virus-Cy3. (**C**) Determination of the viral loads in the whole body and the salivary glands. The nymphs were microinjected with the ds*caspase-1* mixture and infected with RRSV. The RRSV *P8* transcript levels in each nymph (the upper left, one dot represents one insect) and the salivary glands (the upper right) at 8 d p.i. were measured as described in Fig. 1. Western blotting analysis of RRSV capsid P8 protein in the whole bodies (left) and the salivary glands (right) of ds*GFP*- and ds*caspase-1* mixture-treated nymphs is shown in the lower panel. For each treatment, 10 whole bodies or 100 salivary glands were mixed as a sample for analysis. Data represent one of three biological replicate experiments. β-Actin was used to show equal protein loading. (**D**) The silencing of *Nlcaspase-Nc* and *Nlcaspase-8* gene expressions in *N. lugens*. The relative transcript levels of *Nlcaspase-Nc* gene (left) and *Nlcaspase-8* gene (right) after RNAi were analyzed as described above.

RRSV-induced apoptosis occurred at 6–8 d p.i., and so we focused on 8 d p.i. to observe the RNAi effect. We first conducted RNAi experiments by microinjecting *N. lugens* nymphs with a mixture of dsRNAs specific to the three *Nlcaspase-1* genes at a 1:1:1 quality ratio in order to maximally inhibit the expression of all *Nlcaspase-1* genes. Interference notably decreased the expression levels of the three *Nlcaspase-1* genes in *N. lugens* during 2–8 d p.i. compared with ds*GFP*-injected individuals (Fig. 6A). The relative transcript levels of the three *Nlcaspase-1* genes were reduced by approximately 70–90% upon dsRNA injections throughout the tested period, indicating that RNAi sufficiently down-regulated the expression of the target genes. RRSV infection did not cause apoptosis in the salivary glands of ds*caspase-1* mixture-treated nymphs but induced apoptosis in the salivary glands of ds*GFP*-injected nymphs at 8 d p.i. (Fig. 6B). The greenish-yellow fluorescence signals were clearly observed in the nuclear regions of principal gland cells of the RRSV-infected salivary glands. These results suggest that silencing of the three *Nlcaspase-1* genes effectively inhibited apoptosis in the salivary glands of RRSV-infected nymphs, while ds*GFP* treatment did not affect the occurrence of apoptosis at 8 d p.i. Subsequently, we focused on each *Nlcaspase-1* gene to investigate the RNAi effect on RRSV-induced apoptosis in the salivary glands. In each case, the targeted mRNA levels of *Nlcaspase-1a*, *Nlcaspase-1b* or *Nlcaspase-1c* were greatly reduced by approximately 90, 90 and 80%, respectively; while there were no apparent reductions in transcript levels for non-target genes, indicating that the dsRNA-mediated silencing was sequence specific (Fig. 6A). Examining three pools of 200 nymphs each showed that expression inhibition of *Nlcaspase-1a*, *Nlcaspase-1b* or *Nlcaspase-1c* genes sufficiently impeded apoptosis in RRSV-infected salivary glands, suggesting that these *Nlcaspase-1*s were indispensable in RRSV-induced apoptosis (Fig. 6B). These results enabled us to determine whether silencing the expression of the *Nlcaspase-1* genes affected the viral proliferation or accumulation in *N. lugens*, which would result in less or no apoptosis in salivary glands. We tested this by knockdown of expression of all *Nlcaspase-1* genes using microinjection of a mixture of dsRNAs into *N. lugens* nymphs to ensure sufficient inhibition. RRSV was then inoculated into the nymphs, and the resultant viral loads in the whole body and salivary glands of *N. lugens* nymphs were determined using quantitative real-time PCR analysis at 8 d p.i. Among 70 ds*caspase-1*- and 70 ds*GFP*-treated nymphs, 16 (23%) and 15 (21%) individuals were infected by RRSV (Fig. 6C), respectively, indicating a similar infection rate for insects exposed to the different treatments. Viral loads in ds*GFP*- or ds*caspase-1*-injected nymph individuals varied greatly but did not differ significantly between the two groups. Furthermore, we compared the viral loads in the salivary glands of ds*caspase-1*- and ds*GFP*-treated nymphs. As the quantity of salivary glands was very low, we prepared a mixture including 100 salivary glands as one biological sample, and three biological repeats were analyzed. The viral loads in the salivary glands of the ds*caspase-1*-injected nymphs were almost the same as those in the ds*GFP*-infected nymphs, suggesting that RRSV proliferation or accumulation was not significantly correlated with dsRNA treatment (Fig. 6C). To verify the data from the quantitative real-time PCR, western blotting was conducted to determine RRSV capsid P8 protein levels in ds*GFP*- and ds*caspase-1*-treated nymphs. For each treatment, 10 whole bodies or 100 salivary glands were mixed as a sample for analysis. The blotting showed the capsid P8 protein bands were detected in the whole bodies and the salivary glands of the ds*GFP*- and ds*caspase-1*-treated nymphs (Fig. 6C). There were similar signal strengths of the capsid P8 proteins in the whole bodies or the salivary glands between the different treatments. We also investigated the roles of *Nlcaspase-Nc* and *Nlcaspase-8* genes in RRSV-induced apoptosis. Microinjection with ds*caspase-Nc* or ds*caspase-8* led to significant inhibition of expression of target genes. The transcript levels of *Nlcaspase-Nc* and *Nlcaspase-8* were greatly reduced by approximately 92 and 93%, respectively (Fig. 6D). Apoptosis was not observed in RRSV-infected salivary gland cells from the ds*caspase-Nc*-treated nymphs, but was seen in ds*caspase-8*-treated nymphs after examining three pools of 200 nymphs each, suggesting that *Nlcaspase-Nc* was an important factor in RRSV-induced apoptosis; while *Nlcaspse-8* may not have been an indispensable component in the apoptotic response to RRSV infection (Fig. 6B). Although the detailed functional roles of *N. lugens caspases* are yet to be discovered, our findings clearly indicate that the RRSV-associated apoptosis that occurred in the *N. lugens* salivary gland was regulated in a *caspase*-dependent manner.

### Transmission of RRSV from the insect vector to the rice plant

**Figure 7:**
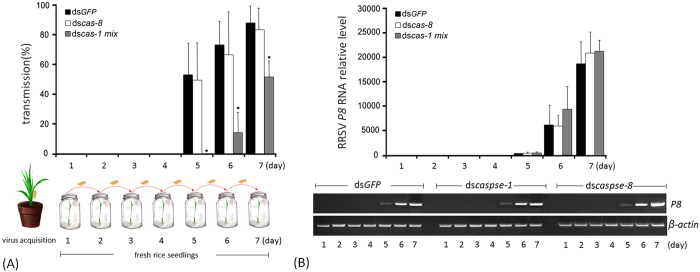
Transmission of RRSV from viruliferous *N. lugens* insects to rice seedlings. (**A**) Second-instar nymphs were microinjected with the ds*caspase-1* mixture or ds*caspase-8* and inoculated on viruliferous rice seedlings, after which they were transferred to fresh rice seedlings as shown in the schematic representation. The ds*GFP*-injected individuals were used as controls. The day on which the insects were transferred to healthy rice seedlings was regarded as the first day and is shown on the *x*-axis. The transmission rate of RRSV from the viruliferous individuals to the fresh rice seedlings is shown on the *y*-axis. Error bars indicate the standard deviations of three independent experiments. The asterisks indicate statistical significance at *p* < 0.05 (*) by Student’s *t*-test compared to the ds*GFP*-treated control. (**B**) Determination of the virus loads after dsRNA treatments in *N. lugens* salivary glands. The *N. lugens* nymphs were treated with ds*caspase-1* mixture, ds*caspase-8* or ds*GFP* and infected with RRSV as described in this figure (**A**). Total RNA was extracted from the salivary glands at the indicated times and subjected to quantitative real-time PCR and reverse transcription-PCR analysis. For quantitative real-time PCR, samples from each time point were tested in three biological replicates, and the mean value used to analyze the relative transcript levels as described in Fig. 1. Reverse transcription-PCR analysis is shown in the lower panel. Total RNA (1 μg) was used as a template. The ds*cas-1 mix*, ds*cas-8* and ds*GFP* refer to ds*caspase-1* mixture, ds*caspase-8* and ds*GFP*-treated samples, respectively.

To understand whether the observed RRSV-induced apoptosis was linked to viral transmission from the insect vector to the rice plant, we silenced the expressions of three *Nlcaspase-1* genes because they were the essential effectors leading to apoptosis. We also silenced the expression of *Nlcaspase-8* gene to understand its relevance to the virus transmission. The ds*caspase-1*-, ds*caspase-8*- or ds*GFP*-microinjected nymphs were inoculated with RRSV and then individually propagated on a series of fresh rice seedlings at 24-h intervals for 7 d (Fig. 7A). In ds*caspase-1*-, ds*caspase-8*- or ds*GFP*-treated group, more than 210 rice seedlings infected with at least 30 viruliferous nymphs were analyzed using reverse transcription-PCR combined with quantitative real-time PCR to detect the virus. In the ds*GFP*-treated group, all tested rice seedlings were virus-negative at 1–4 d p.i., suggesting that virus transmission did not occur from the nymphs to the rice seedlings during this infection stage (Fig. 7A). The virus was detectable in rice seedlings as early as 5 d p.i. The transmission rate from the viruliferous nymphs to the rice seedlings was 53.4% at 5 d p.i. and then increased to 74 and 88% at 6 and 7 d p.i., respectively, implying that the RRSV proliferation in the nymphs promoted viral transmission. In the ds*caspse-8*-treated group, the virus was detectable in the rice seedlings at 5 d p.i. The transmission rate was 50% at 5 d p.i. and then increased to 67 and 83% at 6 and 7 d p.i., respectively. There were no significant differences of the transmission rates between ds*GFP*- and ds*caspase-8*-treated groups during the tested period. In the ds*caspase-1*-treated group, RRSV was not transmitted by the *N. lugens* nymphs to the fresh rice seedlings at 1–5 d p.i. The earliest transmission appeared at 6 d p.i. and, in this case, the transmission rate (15%) was significantly lower than that in the ds*GFP*- and ds*caspse-8*-treated nymphs. Despite the transmission rate increasing to 52% at 7 d p.i., it was still much lower than for controls. We determined the virus loads in the salivary glands of *N. lugens* nymphs after microinjection with ds*caspase-1*, ds*caspse-8* or ds*GFP*. Quantitative real-time PCR analysis showed that *P8* gene expression was not detectable in the salivary glands of nymphs at 1–4 d p.i. but became detectable at 5 d p.i. (Fig. 7B, upper panel). The relative transcript levels were low at 5 d p.i. in ds*GFP*-, ds*caspase-8*- and ds*caspase-1*-treated samples, then quickly increased at 6–7 d p.i. Reverse transcription-PCR analysis detected the weak bands of the amplified *P8* gene produced at 5 d p.i. and the strong bands at 6–7 d p.i. in ds*caspase-1*-, ds*caspase-8*- and ds*GFP*-treated samples (Fig. 7B, lower panel). Although the virus loads were very low in the salivary glands of the nymphs at 5 d p.i., RRSV was effectively transmitted to the rice plant from ds*GFP*- and ds*caspse-8*-treated nymphs, but could not be transmitted from ds*caspase-1*-treated nymphs. With the progress of proliferation, viral loads in the salivary glands of ds*caspse-1*-, ds*caspase-8*- and ds*GFP*-treated nymphs increased rapidly, but transmission efficiency was much lower in ds*caspase-1*-treated compared with ds*GFP*- and ds*caspase-8*-treated nymphs at 6–7 d p.i. These results clearly suggest that the silencing of *Nlcaspase-1* genes caused a significant reduction of RRSV transmission from the insect vector to the rice plants; while the silencing of *Nlcaspase-8* gene did not significantly affect the virus transmission.

## Discussion

The mechanisms underlying virus transmission from the insect vector to the host plant remain largely unknown. In the present study, we examined the biological characteristics of plant virus transmission via investigating insect vector–virus–host plant interactions. Quantitative real-time PCR analysis revealed that RRSV continually propagated in the *N. lugens* salivary glands and reached a maximal accumulation at 12 d p.i. Tracking of the virions through immunofluorescence staining further confirmed the progressive accumulation of RRSV in the salivary glands. TEM and IEM scanning clearly showed that the virions were abundant in the cytoplasm of salivary gland cells, suggesting the proliferation of RRSV in the tissue. The fact that RRSV was not detectable by immunofluorescence staining and quantitative real-time PCR at 2–4 d p.i. was most likely because the virus had not yet spread to the salivary glands during this infection stage. This result is consistent with a report from Wei *et al*.^2^, which showed RRSV infection route in various tissues of *N. lugens*.

Our study revealed that RRSV infection induced apoptosis in *N. lugens* salivary glands. The occurrence of apoptosis was limited to the viruliferous insects, indicating that RRSV accumulation was required to induce apoptosis in the salivary gland cells. Only a portion of the principal gland cells and of the duct epithelium cells of the salivary glands underwent apoptosis upon RRSV infection. Interestingly, the accessory gland cells did not undergo apoptosis, suggesting that virus-associated apoptosis was restricted to a subset of cells within this tissue. Thus the apoptosis that occurred only in principal gland cells and the duct epithelium cells of the salivary glands may be an evolutionary trade-off between the need for RRSV to ensure its release from the insect and the need to ensure its survival in the insect.

Caspases play an important role in triggering apoptosis in animal cells. In this study, we provide direct evidence that caspases were necessary for RRSV-induced apoptosis in *N. lugens* salivary glands. Due to the accomplishments in the *N. lugens* genome project by our research team, we were able to perform a deep search of the genome to identify *caspase* genes. Five *caspase* genes homologous to insect *caspase-1*, *caspase-Nc* and *caspase-8* were identified in the *N. lugens* genome. Our experimental results indicated that the expression inhibition of *N. lugens caspase* genes through *in vivo* RNAi did not lead to phenotypic defects in morphological characters and lethality. However, the silencing of *Nlcaspase-1* and *Nlcaspase-Nc* expression caused the failure of apoptosis in the salivary glands of viruliferous insects. In contrast, RRSV dramatically induced apoptosis in ds*caspase-8*- and ds*GFP*-treated insects. These findings reveal that the molecular mechanisms by which RRSV induced apoptosis in *N. lugens* acted in a caspase-dependent manner. *Nlcaspase-1* and *Nlcaspase-Nc* were critical in RRSV-induced apoptosis pathways, while *Nlcaspase-8* did not seem to be an essential factor during the apoptosis process. The detailed functions of these *Nlcaspase* genes and their interactions in the caspase activation cascades require further elucidation.

As an insect vector, *N. lugens* can effectively transmit RRSV to the rice plant. Successful transmission was achieved by the majority of viruliferous insects that were treated with ds*GFP*. A minority of individuals appeared to exhibit no ability to transmit the virus, most likely due to individual differences, e.g., in the virus loads in the viruliferous insects, in the feeding behaviors of the insect vectors and in the microphysiological or microecological systems of the individual insects. Silencing of *Nlcaspase-8* expression generated the similar transmission rates at 5–7 d p.i. when compared to the ds*GFP*-treated group, suggesting that *Nlcaspase-8* seemed not to be involved in RRSV transmission. However, silencing of all three *Nlcaspase-1* genes significantly interfered with RRSV transmission from the viruliferous insects to the rice plants at 5–6 d p.i., indicating that *Nlcaspase-1*s were associated with virus transmission from *N. lugens* to rice seedlings. The fact that the transmission rate increased to 50% at 7 d p.i. implied that *Nlcaspase-1*s might not have been unique factors in the mechanism of virus transmission. A recent study reported that mosquito saliva serine protease enhances dissemination of dengue virus from vector to the mammalian host^32^. We hypothesize that certain salivary proteins may be conducive to promoting the virus transmission in *N. lugens*. The discovery of RRSV-induced salivary proteins will enable us to better understand the virus transmission mechanism from its insect vector to the plant host.

In this study, our findings established a link between virus-induced apoptosis and virus transmission. This is possibly a strategy of the adaptive evolution of the insect vector with the virus. The connection of RRSV transmission with virus-induced apoptosis in salivary gland cells provides new insights into the specific interactions between the insect vector, virus and plant host. Understanding the characteristics of RRSV transmissibility by *N. lugens* will contribute to the control of diseases caused by insect-mediated plant viruses.

## Methods

### Insects, viruses and rice plants

The *N. lugens* strain employed in this study was originally collected from a rice field on the Huajiachi Campus of Zhejiang University, Hangzhou, China. The insects used in this experiment were the offspring of a single female and were reared at 26 ± 0.5 °C with 50 ± 5% humidity on rice seedlings under a 16:8 h light:dark photoperiod. Newly molted, second-instar *N. lugens* nymphs were used for these experiments. RRSV was kindly provided by Prof. Tai-Yun Wei of the Institute of Plant Virology of Fujian Agricultural and Forestry University and Prof. Xu-Dong Zhu of the China National Rice Research Institute and was maintained at the Molecular Biology Laboratory of the Institute of Insect Sciences of Zhejiang University. The rice TN1 strain, which is susceptible to RRSV infection, was used in assays involving insect-mediated virus infection.

### Analysis of apoptosis

Second-instar *N. lugens* nymphs were inoculated using RRSV-infected rice seedlings for 48 h and then transferred to healthy rice seedlings. The day on which the insects were transferred to the healthy rice seedlings was regarded as the first day of the infection. The nymphs were collected at 48-h intervals during 2–8 d post infection (p.i.); uninfected nymphs were used as the control. For tissue extraction, the nymphs were anesthetized on ice, and their salivary glands were dissected and washed in a phosphate-buffered saline (PBS) solution (137 mM NaCl, 2.68 mM KCl, 8.1 mM Na_2_HPO_4_ and 1.47 mM KH_2_PO_4_ at pH 7.4). Apoptosis was analyzed using the DeadEnd^TM^ Fluorometric TUNEL (terminal deoxynucleotidyl transferase dUTP nick-end labeling) system (Promega, Madison, WI, USA). Briefly, the salivary glands were fixed in 4% paraformaldehyde in PBS (v/v) overnight at 4 °C and washed in PBS at room temperature, followed by incubation with 2% Triton X-100 (Sigma-Aldrich, St. Louis, MO, USA) for 1 h. Then, the salivary glands were permeabilized in 500 μl of 20 μg/ml proteinase K for 5 min and washed in PBS for 5 min. After fixation in 4% paraformaldehyde for 5 min, followed by washing in PBS for 5 min, the tissues were equilibrated in 100 μl of equilibration buffer [200 mM potassium cacodylate (pH6.6), 25 mM Tris-HCl (pH6.6), 0.2 mM DTT, 0.25 mg/ml BSA and 2.5 mM cobalt chloride] for 10 min. Next, 50 μl of a recombinant terminal deoxynucleotidyl transferase reaction mixture was added to the tissues to catalytically incorporate fluorescein-12-dUTP at 3¢-OH DNA ends, followed by incubation for 1 h at 37 °C. After incubation, 500 μl of 2 × SSC was added to the tissues for 10 min, followed by washing three times in PBS (5 min each). Then, the tissues were blocked with 10% fetal bovine serum (Gibco, Grand Island, NY, USA) at room temperature for 1 h. The monoclonal antibody against a major RRSV capsid P8 protein, which was produced in our laboratory, was added at a dilution of 1:200 for 1.5 h and visualized with an Cy3-labeled secondary goat anti-mouse IgG (Abbkine, Redlands, CA, USA), which was diluted at 1:200. For nucleus (DNA)-specific staining, the salivary glands were stained with 100 nM of 4¢,6-diamidino-2-phenylindole (DAPI) (Sigma-Aldrich) for 2 min, after which they were washed three times with PBS. Fluorescence images were examined using a Zeiss LSM780 confocal laser-scanning microscope (Zeiss, Göttingen, Germany).

### Investigation of RRSV proliferation in *N. lugens* salivary glands using quantitative real-time PCR

Second-instar *N. lugens* nymphs were infected with RRSV as described in the previous sections. Total RNA was extracted from the salivary glands of nymphs at 48-h intervals using RNAiso plus (TaKaRa, Dalian, China). The total RNA samples extracted from 100 salivary glands were used as an individual template for quantitative real-time PCR analysis (a total of 300 salivary glands per time point for three biological replicates). The concentration of each RNA sample was adjusted with DEPC-treated H_2_O to 1 μg/μl, and 1 μg of RNA was reverse-transcribed in a 10-μl reaction using ReverTra Ace® qPCR RT Master Mix with the gDNA Remover Kit (ToYoBo, Osaka, Japan) to remove any contaminating genomic DNA. RNA with no-reverse-transcriptase was used as the no-template control (NTC). Quantitative real-time PCR was performed using the BIO-RAD CFX96^TM^ Real-Time System (Bio-Rad, Hercules, CA, USA) and the iQ^TM^ SYBR Green® Supermix Kit (Bio-Rad). The first-strand cDNA and a no-reverse-transcription control were used as templates for three biological replication assays under the following conditions: denaturation at 95 °C for 2 min, followed by 40 cycles of 95 °C for 15 s and 60 °C for 30 s. Fluorescence of PCR products was detected by adding a heat-dissociation protocol (temperature range 65–95 °C) during the last step of each cycle. Following amplification, melting curves were constructed and data analysis was performed on Bio-Rad CFX Manager 2.1 software. One pair of primers (sense: 5¢-GAGATAACGCTTGGAGGACA-3¢ and antisense: 5¢-GGATTGAATTACTCGCAGGA-3¢), that specifically amplify the RRSV *P8* gene encoding the major capsid protein were designed based on the gene sequence (GenBank accession no. HM125546). As an internal control, expression of *N. lugens 18S rRNA* gene (GenBank accession no. JN662398) was analyzed using the following primers: 5¢-CGCTACTACCGATTGAA-3¢ (sense) and 5¢-GGAAACCTTGTTACGACTT-3¢ (antisense). In our previous studies, the utility of the *N. lugens 18S rRNA* gene was validated for its stable expression in *N. lugens* tissues, developmental stages and immune-induced individuals^18^,^20^,^21^. In the present study, the results were standardized to the expression level of *N. lugens 18S rRNA*. The relative transcript levels of RRSV *P8* were calculated using the ∆∆C_t_ method and the following equation: ∆C_t_  = (C_t_ of RRSV *P8* gene) – (C_t_ of *N. lugens 18S rRNA* gene). The biological repeats were analyzed and the average threshold cycle (Ct) value was used to quantify the relative transcript levels of RRSV *P8*.

### Identification of *caspase* genes from the *N. lugens* genomic and transcriptomic databases

The brown planthopper genome assemblies have been deposited at GenBank under accession number AOSB00000000 (BioProject PRJNA177647). The *caspase* genes were searched against the *N. lugens* genome sequence based on the KEGG (ftp://ftp.uniprot.org/pub/databases/uniprot/, v58), Swissprot (ftp://ftp.uniprot.org/pub/databases/uniprot/, release-2012_03) and Trembl (ftp://ftp.uniprot.org/pub/databases/uniprot/, release-2012_03) annotations. Predicted coding sequences of *caspase* genes were used as reference sequences to match the *N. lugens* transcriptome in the Sequence Read Archive (SRA) database (http://www.ncbi.nlm.nih.gov/sra, SRX023419), which were obtained through high-throughput Illumina technology in our previous studies^18^,^21^, using the tBLASTX algorithm with a cut-off E-value of 10^−10^. The deduced CARD and caspase domains were determined by using Pfam (http://www/sanger.ac.uk/Software/Pfam/), Simple Modular Architecture Research Tool (SMART) (http://smart.embl.de/), InterProScan (http://www.ebi.ac.uk/Tools/pfa/iprscan/) and Conserved Domains of the National Center for Biotechnology Information (NCBI) website (http://www.ncbi.nlm.nih.gov/Structure/cdd/wrpsb.cgi).

### Phylogenetic analysis

The *N. lugens caspase-1a*, *caspase-1b*, *caspase-1c*, *caspase-Nc* and *caspase-8* sequences were aligned with the best-matched homologs of other insect species using the ClustalX program^33^. The phylogenetic trees were constructed by the maximum likelihood method using the program Mega 5.05 (http://www.megasoftware.net/)^34^. Homologous relationships were determined using bootstrap analysis with 1000 replications.

### Preparation of double-stranded RNA (dsRNA)

Nucleotide sequences specific to the *N. lugens caspase-1a*, *caspase-1b*, *caspase-1c*, *caspase-Nc* and *caspase-8* were individually cloned into the pGEM-T Easy vector (Promega). *Aequorea victoria* green fluorescent protein (*GFP*) was used as a control. Specific dsRNAs were synthesized via *in vitro* transcription using PCR-generated DNA templates that contained the T7 promoter sequence at both ends. The specific primers used to generate these DNA templates are shown in a supplementary file 1. The specific dsRNAs for each gene were synthesized using the MEGAscript T7 Transcription Kit (Ambion, Austin, TX) according to the manufacturer’s instructions. Following transcription, the DNA template was removed using TURBO DNase (Ambion) and the dsRNAs were purified with the RNAqueous Kit (Ambion). The size of the dsRNA products was confirmed by electrophoresis on a 1% agarose gel that was run in Tris-Acetate-EDTA buffer.

### RNA interference (RNAi) via microinjection

Second-instar *N. lugens* nymphs were used in the RNAi experiments. Each virus-free nymph was anesthetized with carbon dioxide (CO_2_) for 5–10 s at P_CO2_ = 5 mPa. Approximately 250 ng of dsRNA was microinjected into the thorax of each nymph between the mesocoxa and the hind coxa using the FemtoJet Microinjection System (Eppendorf, North America). The treated nymphs were reared at 26 ± 0.5 °C with 50 ± 5% humidity on fresh, healthy rice seedlings under a 16:8 h light:dark photoperiod for 24 h and then transferred to the RRSV-infected rice seedlings for 48 h. The morphological phenotypes and mortality rates of the insects were observed and determined using a stereomicroscope (Leica S8AP0, Germany) every 24 h following the dsRNA treatments.

### Confirmation of RRSV infection in the salivary glands using immunofluorescence staining

Second-instar *N. lugens* nymphs were inoculated on RRSV-infected rice seedlings for 48 h and then transferred to healthy rice seedlings. The salivary glands were dissected from the *N. lugens* nymphs at 48-h intervals, and uninfected nymphs were used as controls. The salivary gland samples were fixed using 4% paraformaldehyde in PBS for 6 h at 4 °C and then blocked with 10% fetal bovine serum (Gibco) at room temperature for 2 h. A monoclonal antibody against a major RRSV capsid P8 protein was diluted 1:200, followed by overnight incubation at 4 °C and visualization using a FITC-labeled secondary goat anti-mouse IgG (Jackson ImmunoResearch, West Grove, PA, USA), diluted 1:100. After the subsequent washes, the tissues were stained using 100 nM DAPI (Sigma-Aldrich) for 2 min at room temperature. Following three washes with PBS, each for 10 min, fluorescence images were observed using a Zeiss LSM 780 confocal microscope (Zeiss).

### Transmission electron microscopy (TEM) observations

Second-instar *N. lugens* nymphs were infected with RRSV as described in the previous sections. The nymphs were collected at 8 d p.i.; uninfected nymphs were used as a control. The salivary glands were dissected from the nymphs and fixed in 2.5% (v/v) glutaraldehyde in PBS at 4 °C overnight. Following fixation, the samples were washed three times in 0.1 M PBS (pH 7.0) and then post-fixed with 1% (v/v) osmium tetroxide for 1 h at room temperature. The fixed salivary glands were dehydrated through incubation in a graded series of ethanol (50, 70, 80, 90, 95 and 100%, v/v) for 10 min each and then soaked in acetone for 20 min. At room temperature, the samples were placed in a 1:1 mixture of acetone and Spurr resin for 1 h, then in a 1:3 mixture for 3 h and in Spurr resin alone overnight. Finally, the samples were polymerized at 70 °C for 16 h. Semi-thin sections (2 μm) were cut using glass knives on an LKB Bromma 11800 pyramitome (LKB, Bromma, Sweden) and stained with methylene blue, and ultra-thin sections were cut with a diamond knife using PowerTome-PC (RMC, Boeckeler Instruments, Tucson, AZ, USA). The sections were stained with 3% uranyl acetate and alkaline lead citrate and observed using TEM with a model JEM-1230 (JEOL, Tokyo Japan) at an accelerating voltage of 80 kV.

### Immunoelectron microscopy (IEM)

As described in the previous sections, RRSV-infected *N. lugens* nymphs were collected at 8 d p.i.; and uninfected nymphs were used as a control. Salivary glands were dissected and fixed in 4% paraformaldehyde (v/v), 0.3% glutaraldehyde and 4% sucrose in 0.1 M sodium cacodylate buffer (pH 7.4) for 3 h. After dehydration in serially graded methanol (30, 50, 70, 80, 90, 95 and 100%, v/v), the tissues were embedded in Lowicryl K4M (Polysciences, Inc., Warrington, PA, USA). Polymerization was performed in an ultraviolet irradiator at −20 °C for 2 d and then at room temperature for 2 d. Ultra-thin sections were cut as described for the TEM observations. The sections were blocked with 1% BSA for 5 min and then incubated with the anti-RRSV capsid P8 protein mouse serum (1:50) at room temperature for 2 h, followed by incubation with 5-nm gold-conjugated goat-anti-mouse IgG (1:200, Sigma-Aldrich) for 2 h. The sections were stained in 3% uranyl acetate (w/v in distilled water) and observed using TEM with a model JEM-1230 at an accelerating voltage of 80 kV.

### Western blotting analysis

Salivary glands and whole bodies were collected from *N. lugens* nymphs and homogenized in RIPA Lysis Buffer (Beyotime, Shanghai, China), respectively. The protein concentrations were quantified using Pierce BCA Protein Assay Kit (Thermo Scientific, Rockford, IL, USA) following the manufacturer’s instructions. After adding 6 × SDS loading buffer, lysates were boiled for 10 min. The proteins were separated by SDS-PAGE and transferred to PVDF membrane. The blot was probed with a RRSV capsid P8-specific mouse primary antibody (1:5,000 dilution) and detection was achieved using a goat anti-mouse IgG-conjugated horseradish peroxidase (HRP) antibody (Jackson ImmunoResearch, West Grove, PA, USA) at a dilution of 1:10,000. Western blots were imaged using a Chemiluminescence Detection Kit (Bio-Rad, Hercules, CA, USA) with the Molecular Imager® ChemiDoc™ XRS+ System (Bio-Rad, Hercules, CA, USA). The β-actin polyclonal rabbit serum was prepared as in our previous report^35^, and used to monitor equal protein loading.

### Transmission efficiency of RRSV from the insect vector to the rice plants

Second-instar *N. lugens* nymphs were microinjected with a mixture of ds*caspase-1* at a 1:1:1 quality ratio or ds*caspase-8* and maintained on healthy rice seedlings for 24 h. The dsRNA-treated nymphs were placed on RRSV-infected plants for virus acquisition over an acquisition access period (AAP) of 48 h. Each nymph was individually transferred to a series of fresh, virus-free rice seedlings at the 1–2 leaf stage in culture bottles (one nymph per bottle) and reared at 26 ± 0.5 °C with 50 ± 5% humidity under a 16:8 h light:dark photoperiod. Two weeks later, the transmission of RRSV from the insects to the rice seedlings was determined using quantitative real-time PCR combined with reverse transcription-PCR. Briefly, total RNA was extracted from the insects and the leaf tissue of the rice seedlings using RNAiso plus (TaKaRa) and employed as the templates for reverse transcription. PCR analysis was performed using the specific primers to amplify the RRSV *P8* gene encoding the major capsid protein. The transmission rates of RRSV to the rice seedlings by the viruliferous individuals of *N. lugens* were calculated for 1–7 d p.i. based on the PCR results. The ds*GFP*-microinjected individuals were used as controls. RRSV accumulation after dsRNA treatments in *N. lugens* salivary glands was investigated using quantitative real-time PCR and reverse transcription-PCR. Total RNA was extracted from the salivary glands of ds*caspase*-*1*-, ds*caspase*-*8*- or ds*GFP*-treated *N. lugens* nymphs at 24-h intervals during 1–7 d p.i. Reverse transcription was conducted using 1 μg of genomic DNA-removed RNA sample. The procedure for quantitative real-time PCR analysis is described in Materials and Methods: ‘Investigation of RRSV proliferation in *N. lugens* salivary glands using quantitative real-time PCR’. Reverse transcription-PCR was carried out using a pair of primers for amplifying the RRSV *P8* gene (sense: 5¢-GAGCAAACTTGAGGCGTAT-3¢ and antisense: 5¢-TTGGTCGTGTTGTATCTGG-3¢). The *N. lugens β-actin* gene (GenBank accession no. EU179846) was analyzed as a control using the following primers: 5¢-TGGACTTCGAGCAGGAAATGG-3¢ (sense) and 5¢-ACGTCGCACTTCATGATCGAG-3¢ (antisense). The PCR reaction was performed under the following conditions: denaturation at 94 °C for 4 min followed by 35 cycles of 94 °C for 30 s, 60 °C for 30 s and then extension at 72 °C for 10 min.

## Additional Information

**How to cite this article**: Huang, H.-J. *et al*. Rice ragged stunt virus-induced apoptosis affects virus transmission from its insect vector, the brown planthopper to the rice plant. *Sci. Rep*. **5**, 11413; doi: 10.1038/srep11413 (2015).

## Supplementary Material

Supplementary Information
