# Supplementary material for: Rice ragged stunt virus-induced apoptosis affects virus transmission from its insect vector, the brown planthopper to the rice plant
Source: Sci Rep. 2015 Jun 15;5:11413. doi: 10.1038/srep11413 (PMC4466780; doi:10.1038/srep11413)
Supplement: Supplementary Information [file srep11413-s1.doc]

#### Rice ragged stunt virus-induced apoptosis affects virus transmission from its insect vector, the brown planthopper to the rice plant

Hai-Jian Huang^1^, Yan-Yuan Bao^1^*, Shu-Hua Lao^1^, Xiao-Hui Huang^1^, Yi-Zhou Ye^1^, Jian-Xiang Wu^2^, Hai-Jun Xu^1^, Xue-Ping Zhou^2^, Chuan-Xi Zhang^1^

*^1^State Key Laboratory of Rice Biology and Ministry of Agriculture Key Laboratory of Agricultural Entomology, Institute of Insect Sciences, Zhejiang University, Hangzhou 310058, China*

*^2^ Institute of Biotechnology, Zhejiang University*

* Correspondence to Y.-Y. Bao ([yybao@zju.edu.cn](mailto:yybao@zju.edu.cn))

Supplementary file 1: Table 1 Primers used in double stranded RNA synthesis and quantitative real-time PCR

| *Caspase* genes | Forward primer (5’-3’) | Reverse primer (5’-3’) |
| --- | --- | --- |
| Primers used in double stranded RNA synthesis | | |
| *Nlcaspase-1a* | TAATACGACTCACTATAGGGAAGCAAATGGGAGTGATGAACC | TAATACGACTCACTATAGGGAATAAGCGATAAGGAAGTCGGC |
| *Nlcaspase-1b* | TAATACGACTCACTATAGGGGCAGATCATTCAAAACGTGAAGG | TAATACGACTCACTATAGGGCGAGCCTTTTTCTTCGTTGCG |
| *Nlcaspase-1c* | TAATACGACTCACTATAGGGGAAGTGCGCCAGAGTGTACAAC | TAATACGACTCACTATAGGGCTTTCTCCGGGTTTCGCCATG |
| *Nlcaspase-Nc* | TAATACGACTCACTATAGGGTCATGGAATCCAAGGACATAG | TAATACGACTCACTATAGGGTGAATTCGTTCAGTTTATCGC |
| *Nlcaspase-8* | TAATACGACTCACTATAGGGGAGTTGTAAGCGAGAACTTAC | TAATACGACTCACTATAGGGCGAACCTTCTCTGGTATGACG |
| *AvGFP* | TAATACGACTCACTATAGGGAGAATGAGTAAAGGAGAAGAACTTTTC | TAATACGACTCACTATAGGGAGATTTGTATAGTTCATCCATGCCATGT |
| Primers used in quantitative real-time PCR | | |
| *Nlcaspase-1a* | GAGGCAAAGAGTCCAGTAGCA | AACCGAGTCTCGTCAAAGTGT |
| *Nlcaspase-1b* | TATTTTGTTGGAAGTCTGTG | TTCGTTTAGTCTTGATGTTA |
| *Nlcaspase-1c* | GTGAGGTGCTAGAGGAGGAAGG | TTGAAGGTCTGTTTCGGGTTG |
| *Nlcaspase-Nc* | GCGACTGGGTTAAAGGAAGCT | CGCAGGTCATAAATGGAGGAG |
| *Nlcaspase-8* | GTGCTTTTCGTCAGTTAGTGG | ACGAGCTTGTTTCTGACACCT |
| *Nl18S rRNA* | CGCTACTACCGATTGAA | GGAAACCTTGTTACGACTT |

Supplementary file 2: List 1 Nucleotide and deduced amino acid sequences of *N. lugens caspase* genes

***Nlcaspase-1a***

‍TGCTTGGATGACGTCAATTGCCAAATAAATATTTTTCTTTGTGTTTGTAATAATAAAATA‍AGTTCGTACTTTTAGTCGTACTTTCAAATTGTAAAAAGTCTTTTCCTTCTTTGAGTATAT‍TTGATTGACAGTTGTTTTTGTGTCGTAATCTTGAAAACTTGTGACCACAGCAAAATTTTT‍AACAAAGACTTTTTTTCCAATTACGCTTGCTGAAATTTGTTTTTTCACAAAATGGAAACT‍GATAAATCGAAATCTGAAGCAAATGGGAGTGATGAACCTGACGTTTTTCCATTTTCAAAT‍AATGATGCCAGCAATAAGAACGAGGCAAAGAGTCCAGTAGCAAAAGAATCCTTATTCTAC‍AACATGAACCATAAAAGTCGTGGCCTAGCAATCATTTTCAATCATGAGAAATTTGATAGT‍GATTCTTTGAAACAAAGAAATGGCACAAATGTAGATGCTGACAACCTAAAAAACACTTTG‍ACGAGACTCGGTTTTTCTGTTGCCGTGTTCAAAGACTACTTATGCCATCAAGTTGAGAAT‍GTACTCATTGAAGCAAGTAAGGAGGATCATTCCGACTTCGATTGTTTCGTAGTTGCTGTG‍TTGAGTCATGGAGAACATGGAATTATATACGCTAAAGACCATGCTTATAAACCGGAAATT‍CTATGGACAAAATTCTCTGCAGACAACTGTAAAACACTGGCAGGGAAACCGAAGTTGTTT‍TTTATCCAGGCATGTCAAGGTGATAAATTGGATGGTGGAATCAAGATGACTCAAACTGAT‍TCCAGCTCGACTTATAAAATTCCAGTACATGCCGACTTCCTTATCGCTTATTCAACCATC‍CCTCAGTACTATTCGTGGCGCAACACAGCCCGAGGCTCGTGGTTCGTGCAGGCGCTGTGC‍AGTGTGCTCGACGAAATCGGCACCGACTACGACATACTCACCCTCTTGACGCTGGTCAAT‍CAGCGTGTGGCCTTCGACTTTGAATCCTATGTTCCCAACGATAGTAGGATGCATGCTCAA‍AAGCAAATTCCTTGCATCACGTTCATGCTCACCCGCCTCTTGAAGTTCAACATAAAGACT‍CAACCAATGCCAACATAATTATATTAGCAACTAATCGGGTTTCTAAGACTATGTGCAAAG‍TGAGAAAGTAATAGAAGTGAAATTCTAATGTGATGAAAGCATAGTTGCCTTCAAAACTAT‍ATAACAGTCAATATATTTGACAAGTCCAAGGCCCGGTTTCACTAAAGCCTGTTAAAATTG‍ATTCATTGTTAAATTCTATGAGAACCAAACAGAAAAGGCTTTTCTGAGAAGACTACTTCT‍CTGATTGGCTCTCGTGGCATTTGATCGGGATTAAAAGTTATCAGACTTCTGTGCAACCGG‍TGAAGCTTACTGTTTTGTAAAAGTGTTCAATTGAT‍

**Amino acid sequence (288aa)**

METDKSKSEANGSDEPDVFPFSNNDASNKNEAKSPVAKESLFYNMNHKSRGLAIIFNHEKFDSDSLKQRNGTNVDADNLKNTLTRLGFSVAVFKDYLCHQVENVLIEASKEDHSDFDCFVVAVLSHGEHGIIYAKDHAYKPEILWTKFSADNCKTLAGKPKLFFIQACQGDKLDGGIKMTQTDSSSTYKIPVHADFLIAYSTIPQYYSWRNTARGSWFVQALCSVLDEIGTDYDILTLLTLVNQRVAFDFESYVPNDSRMHAQKQIPCITFMLTRLLKFNIKTQPMPT*

***Nlcaspase-1b***

CTGACAGCTTAGAATTTGTTGGAGGCCTTGGAGCTTGTTGCTTTGAGGTTTGATTGTAAG‍CTGTGAGTGAAAAAATGGAAGCAGATCATTCAAAACGTGAAGGAATTAGGGAAGATGAGC‍TCGATAGTCTAGAAGCAAATGCGACCTACAACATGAACCACAAAGCACGTGGCTCAGCGA‍TCATTTTCAATCACGAACATTTTCTTGAAAATTTGGATCAAAGAAAAGGTACAGATGTTG‍ATGCTTTCAAACTGCGAAATTCTTTAGAAAGACTAGGGTTTTCTGTTGTCTCATATAAAG‍ACCGCAAAGCAGAAGAAATTGAGAACATAATCACTGAAGCAAGTGAGGTTGATCATTCAG‍AGTATGACTGTTTTGTGGTAGCTATATTGAGTCATGGAGGAAATGGATATATAAACGCAT‍ATGATCATCCTTATAAACCAGATATTTTGTGGACCAAGTTTTCTGCAGACAATTGCAAAA‍CACTTGTTGGGAAACCGAAACTCTTCTTCATCCAGGCTTGTCAAGGAAGTGAATTAGATA‍ACGGAATTACTCTATCGGTTCAAACTGATTCCAGTTCCACAAATGAATTTTCATTTCATG‍AAGAAGACTTCCTTATTGCTAATTCGTCTATCCCTGGCTTCTTTTCATTTCGCAACGAAG‍AAAAAGGCTCGTATTTTGTTGGAAGTCTGTGCAGTATGCTCGACAAGAGAGGCACTGACT‍ACGATATATTGACACTTATGACAATGGTCAATCACCATGTGGCTATCTACTTCGAATCCA‍ATAGTGAAGAAGAAATGTATCATGCTAAAAAACAAATTCCTTGTATACATTCCACACTCA‍CCCGCCGATTGAAGTTTAACATCAAGACTAAACGAATTACAAC‍AGAATGATATTATAAATAATCGTTTTTTTCAATCACGATTATCTTGTCAAGTGAGAAAGTAATGAAACTGAGAATCTAATGTGATTTTTG

**Amino acid sequence (271aa)**

MEADHSKREGIREDELDSLEANATYNMNHKARGSAIIFNHEHFLENLDQRKGTDVDAFKLRNSLERLGFSVVSYKDRKAEEIENIITEASEVDHSEYDCFVVAILSHGGNGYINAYDHPYKPDILWTKFSADNCKTLVGKPKLFFIQACQGSELDNGITLSVQTDSSSTNEFSFHEEDFLIANSSIPGFFSFRNEEKGSYFVGSLCSMLDKRGTDYDILTLMTMVNHHVAIYFESNSEEEMYHAKKQIPCIHSTLTRRLKFNIKTKRITTE*

***Nlcaspase-1c***

GTGGCTGAACTTTGTCTCAGATGCTATGAATCCATCAACCGATTCTGATAACTTTGAGAT‍GTCGGTCGGCAGCAAGGAGCAATCGGCGATGGAGCGAGTGGAGGAGAGTGACAACTCTGG‍CCTCCTCTTCGAGGGAGACGACGAGGTGTTCGACGAGGCCGAGGACTTCCCTCGCCAGCC‍GCGGACGCCCAGGGTGCGACGCATATCCGACGTCATCGACTCGATCGGAGAGAGTCCGTC‍GCATCTCACCAAAGACAGGACATACACGTTTCCTACCATCGAAAATTCTTCGACTTACTA‍CTCGGCCATGACTGAGTTTAAGTTGCAGAGTCCGCCCCGCCCCGGGTCGACAGTGCCCGA‍CGGCCTGATGTCGCCGCCGCAGACTCCCGGCTCCGATCCGGGCTACATGAGCACTGGCAC‍CCCGACCACCCCCTCCACTCTGTATCTGCGCCGCAAGGCGTTTCGCTTCAGTGCGTCCAG‍TGTGGACATGGCCGGATCGCCGCCTCGTCACTGGGACGCCACGCAGATCGACGCCAAGGC‍GTTTGCCAATCAAACACAGTTTGAACAACCAAGTGTTGTAGTAAGCCCACCACTGCCCCA‍ATGGCCGCCGAGAAGTGCGCCAGAGTGTACAACATGAACCACAAGAAGAGAGGCCGCACT‍CTCATATTCAACCACGAGGAGTTCACCGACATGCCGGCACGCGATGGATCCGGCATCGAC‍GTCAAGAGACTGGAATGCGCATTCAAAAGTCTCGACTTTGAAGTCGATGTCTATCAAGAT‍CTCAAGGTCGAGAAGCTCAAGGAAGTTATCAACGAAGTGTCACTAGGAGACCATAAGGAC‍GAGGACTGTCTAGTGGTGATTGTGCTGACCCATGGTCTGGGCAACGGAATGCTCTTCGCC‍AGAGACTATGCCTACCCGGTTGAACACCTGTGGGCTCCTTTTGCCGGCGATAAATGCCTC‍AGTCTCGCTGGAAAACCCAAATTGTTCTTTATACAGGCCTGTCGCGGTGAAAAACTTGAT‍GGAGGTCTCACCCTTGTCAATTGTACACAAACTGATAACAACCTCTCGAGCTACAAAATA‍CCATCAATGGCTGATTTTCTCCTGGCTTTCAGCACTTTTGAAGGACATTACTCATGGCGA‍AACCCGGAGAAAGGCACCTGGTTCATCCAGGCGTTGTGTGAGGTGCTAGAGGAGGAAGGC‍ACGAAAAGCAGTCTGCAGGAGATCCTGCTGGAAGTGTCGCGTCGCGTCGCCACCAATCAC‍GAGAGCTACAACGACATGATCGCCTGGCAACACCAGAAGAAGCAGGTGCCTCAGATCAAC‍TCCACCCTCTTGCGTCAGGTCTACTTCAACCCGAAACAGACCTTCAACGAAGATTAGAGG‍TTACTGCCTTTAGGTAAATTTCACAACAGTTTTGAGAACGAGTCTTTTCAGAAGCCCAG‍ACTACTCTGCAG

**Amino acid sequence (258aa)**

MAAEKCARVYNMNHKKRGRTLIFNHEEFTDMPARDGSGIDVKRLECAFKSLDFEVDVYQDLKVEKLKEVINEVSLGDHKDEDCLVVIVLTHGLGNGMLFARDYAYPVEHLWAPFAGDKCLSLAGKPKLFFIQACRGEKLDGGLTLVNCTQTDNNLSSYKIPSMADFLLAFSTFEGHYSWRNPEKGTWFIQALCEVLEEEGTKSSLQEILLEVSRRVATNHESYNDMIAWQHQKKQVPQINSTLLRQVYFNPKQTFNED*

***Nlcaspase-8***

CTGTGTATTTGCTCTTATCACAATATAATCATACAAAAAGTGGTTTGTTTTCAATTTTATTATAAGTAGCATGGTGTACTTTAAAAATGGAATAAGTTTAACGATTGTATTATGAAACTAGATTTATTAGTAAGTACTAATGCTTTTAGTAAGTTTGAGTTTCATATTATTTATTTTAACTAGTAGAGTGGAAGTGACAAGGAAAACCAAAGTTTTTTATTATTGAATAAGATTATGGTGAGATGGATCCACACAATGAAATAAGCAGTACTCATCTACAAGATCCAAGTGGAAAACAGATGGACTACCCTTCACATGGAGGAAAATCGCAGGAAAATTATTGTTCAATCCGTTTCAGTCAGGAAAAACTGACAGAAAATATCACCTTGGATGTCATAGAACTTGTGGAGAAAGACATGGACTTTAACGATATTGTGTCTCTGTTGTTTCTTGTCAGTGAAGAACAACATTCCAAGTATATTTTTCAGCGCATCGCTCAATTCATCAAAATCAAATCAGCAAACCTTGATAGTTTAGAAAGTGTGAACTACAGTCTTCTTTTGGATTGGTGCTTATCTAATCCAAAGAACTGGCGGATGAAAATTGTTGAAGCTCTAGGGATTATTGAGTGCTTCGATATACTCTCAAAACTGGGCTACAAGAAAATAGATGTTATGGAGCACTTCTTACCGGATGATACTGAGTTCGCCATATGCATTCCACTACTAAAAAAGAAACTCTACATTGAGTTATCAAACTCTCCAATCTCGGAAATTTTGTCCCTCTATGAGTGTCTAGAAAAGGATGGTGAAAATTCGAATGATATATTGTTCCAAGATTCATTTATAAATAAGAACTACTTTGAGTTTCTGCTCATTTATCTGTCATCGGAAACATTGGTCAGATTTGACGATCATCCAGACTTGAAGTTTTTCATAAATTTACTTGAAAAAGCAAACTTGCTCAAATTGAAACATATATTTGAAAATTATTTATCGGAACATGAGAATTTAAGTGAACATTGTAAAACAAATGAAAACTTGAGCGAGTCTTCTCCTGGATATAATAAGCCATACACTAAAGACTCTTCCAGTATAGATAAAATGGGCAAAGAGGAATTTGGAAGATTAGGAAACGCAGAAAGGATAGCTGTTGCTCATGACGATTCTGAATTGAGCTATTACAAAATTGGCAATCCCGAGGATCTAGGCATTTGTCTGATAATCAACCAGAAGAATTTCACAAGATTAAGAGAGAGAGATCCTAAATATCAGAGAGTTGTAAGCGAGAACTTACTAGTGGACCGACATGGCACTGAAAGAGACGTTGAGAGGTTGCAGGAAACTTTCAAGCATTTCAAAGTTTCAGTTATTGTTGAGAATGACGTGCCCCACTCGATGATTGGCAGTTGTATAAAAGACACAATAGATACCGATTTCAAGCAACACCATTCGGTATTTTTCTTAGTCATCCTGTCACATGGAGATCAAGGTATTATCTACGGAGTTGACAGTATACCAATTGCTATAAGTTCGCTTGTTGATGCAGTGCTAAGATCATATGATAAACTAAAAAACATACCCAAAGTCATTATTGTTCAAGCCTGTCAAGGAAACTTGCCAAACCCAGTTTTGGAAACTGACGGAGGAAGCTGTGCAGAAACAGTTGCTACTCAACCCAAGTACACTGCGATTGGTTCTGAAAAGCAGGACAAAAAACAGGAGAAAGTTCGGACCGCCTACCATGACGATTTGTTGATGTGCTTTTCGTCAGTTAGTGGATATGAATCTTACCGTCATACCAGAGAAGGTTCGAAATACATTCAAATTCTATGCGATAATTTGATGAAATATGGACACAACGATGACTTTCTGAGCATATGCACACGAGTCAATAATGATCTCAAGAAGCTGTATGTTCCTATCAAAATTGATGATAATGTACTATTGCCATCAACACAGGTGTCAGAAACAAGCTCGTGTCTACATAAGAAACTGTTTTTAGTACAACCATGGAGATCCAATGTTCCAACGTATACTAACAGACCAATAAGTTGAAATAAATTTCTAAAAATTGATCTAGTATCTGTTATTTACTTGATTCACACTTCTGTATGCGTATTTAGTTGAAAATGAAGCTTGATGTACTTCCAAATTCTAAAAATCGATTTTATACTATTGGGTACAGTATAAAATAGTAATATACTATTGTGGCTATTTCATTTAAATACAAGAACAGATCATGTACAATGTTTAGGAAAGGACTACAGGCTGAG

**Amino acid sequence (604aa)**

MDPHNEISSTHLQDPSGKQMDYPSHGGKSQENYCSIRFSQEKLTENITLDVIELVEKDMDFNDIVSLLFLVSEEQHSKYIFQRIAQFIKIKSANLDSLESVNYSLLLDWCLSNPKNWRMKIVEALGIIECFDILSKLGYKKIDVMEHFLPDDTEFAICIPLLKKKLYIELSNSPISEILSLYECLEKDGENSNDILFQDSFINKNYFEFLLIYLSSETLVRFDDHPDLKFFINLLEKANLLKLKHIFENYLSEHENLSEHCKTNENLSESSPGYNKPYTKDSSSIDKMGKEEFGRLGNAERIAVAHDDSELSYYKIGNPEDLGICLIINQKNFTRLRERDPKYQRVVSENLLVDRHGTERDVERLQETFKHFKVSVIVENDVPHSMIGSCIKDTIDTDFKQHHSVFFLVILSHGDQGIIYGVDSIPIAISSLVDAVLRSYDKLKNIPKVIIVQACQGNLPNPVLETDGGSCAETVATQPKYTAIGSEKQDKKQEKVRTAYHDDLLMCFSSVSGYESYRHTREGSKYIQILCDNLMKYGHNDDFLSICTRVNNDLKKLYVPIKIDDNVLLPSTQVSETSSCLHKKLFLVQPWRSNVPTYTNRPIS*

***Nlcaspase-Nc***

GTTATCAATTGCTCTCTGCTCTGGTGAATAAAAGTTTGAAGATACATAAA‍TAAGTTTATTTTGTTTGAATAAGTGAACTTTTTAATTGTTTTCTGCATTC‍TAGCACACTCGTCTATCTCTAGGTAGATGTAAACGGTAACATCATGGAAT‍CCAAGGACATAGAATTGTTGACAACGCATATTGATTTATTGATGAAAGCT‍AATAACGTTGATGCTCTCCTAAATGAGTTGCTTGATAGAAAAGTACTGTC‍TGAGTACATTGCTTATCAGTTGAAAATGATTCCAAACGAAGAGATAAGAC‍TCAAAACTTTATTCGAGGAACTTAAAAGTCGTGGCCCGGCGGCCTTTGAG‍CACTTGCTAAGTTCTCTGGTATCTACTGGCAATGAAGATATTTGCGATAA‍ACTGAACGAATTCAAAAATCCGTCTTCTGTATACTCCAAAATCAAAGTCA‍TAAAAGCAGTGACCCCTCTTGATGTAGAAGATGGTTCATCGATGAAAATC‍TACCGTATGCGGTCGTCACCCAGAGGCTACGCGCTCATAATAAACATCCA‍CGAATTCTTCGAGCCGATTAAGTCATCTGATGACGACACCAGGCATGGCT‍CATACAGGGATGTGGCAAACCTCTACTCTCTCTTTTCCCAGCTAGGTTAC‍AAAGTTAGGATGCACTACAACCTCACAAAAAAGGAAATACACGACAAAGT‍GAGCAGTTTTGCCGAGATGCCGGAGCACGCCTCTGTCGATTCAACAGTGG‍TTGTGGTGATGAGCCATGCAGGGAGATATCATGACACATTTCGGTCCTAT‍GACAATCTACCAGTTCACTGCGACAAGGATGTAGTGGCGCATTTCACCAA‍TCACAAGTCAGAGCACCTCCATGGCAAGCCAAAAATATTCATTTTTCAAA‍TCTGCCGTGGAAATCAACGTGACCATGGAAAACGTGTTGAAAAAACTTTA‍CCGTCAACTACTCAAGACATAGGTGAAAAATCAACTAGTGACGCAACTGG‍TTCGTCAACTGTTGGCAAAACTAGCTCATCAACTGTTGTTGAACCTGGTC‍TATCAACTAGTTTATCAACTGTTGATGTAACTGGTTTATCAACAACTGTT‍GATAGAACTAGTTCTGATGCGTTTCGTTGGGCAACTGGTAATTCAACTAC‍TTCATCAGATGGCAGAATATTCGAATTGCCAAACTACGATGAACCAGTTG‍ATCAACCACAATCAGCTGGCCGGCAACGTCAGTCAACTGTAACTGTAACC‍CGTGAAGCGAGGGTTAACAACATCTTCAAAATCTATTCATCTACTTTTGA‍TTATCAGTCAATTCGCGACTGGGTTAAAGGAAGCTACTTTGTGGATATTA‍TGTGTCAGATCTTCGCTGCAGAAGCCCACAGGTTGGAACTGCGTGATTTG‍CTGCATGAAACTGCCAATGTTGTCTCCTCCATTTATGACCTGCGCTACGG‍CATCATGACGCCGAGCATCACCTGTTCGGCAGACTTTGGCAAACTCTACT‍TCAATCCTGGAATTGACGATGCAACCACCGTTGTAATTCATCAAGTCTCC‍GACAGGAATGATGGCGACTCTAGTGGGGGAAACACTTCTGACAATTGTGA‍CAGAATTGATTACTCTAACAGAAATTATGGTAACTCTGGTGAAAGAAACA‍CTCCTGACAATGGCGACAGAATTGATGAATCAGACAGAATTAATGGCAAT‍TGTAGAGAAGATACTGAAAGAAATGTTTCCAACAGAAATGATGACAAGTG‍TAGAGAAGATGGTGAGAGAAATGTTTGTGATAATGGTGATAGAACTGATG‍ATTCCAACAAAGAAATTTCTAACTATAGAGAAGATTGTGATAGAAATGTT‍TCTAACACTGGTGATAGAATTGATGAGACTAACAGAAATCATGGCAACTG‍TAGAGAAGATACTGAAATTAACTCTTCCAACAACAGTGAAGAAAACACAA‍GTCATTCAAGTGACGAAAATGTTCCCAATCATATTGATAGAAACAATGAA‍AACCCAAGTGAGTTTAGAGACATAAACAGTAGCAGTTATAATGACGCAAT‍CGTTGACAATAGCAGTGTCCCAAACAATAGTAACAGAAATACTAGAAACA‍ATAGTGATTATGATGAAGCCATTGACACGGTTGATGAAGCTATTGATTAT‍AGAGAAATTAACAATAGTAGGTATGATGAAAAACTTGTTGACAATACCAG‍TT‍

**Amino acid sequence (686aa)**

MESKDIELLTTHIDLLMKANNVDALLNELLDRKVLSEYIAYQLKMIPNEEIRLKTLFEELKSRGPAAFEHLLSSLVSTGNEDICDKLNEFKNPSSVYSKIKVIKAVTPLDVEDGSSMKIYRMRSSPRGYALIINIHEFFEPIKSSDDDTRHGSYRDVANLYSLFSQLGYKVRMHYNLTKKEIHDKVSSFAEMPEHASVDSTVVVVMSHAGRYHDTFRSYDNLPVHCDKDVVAHFTNHKSEHLHGKPKIFIFQICRGNQRDHGKRVEKTLPSTTQDIGEKSTSDATGSSTVGKTSSSTVVEPGLSTSLSTVDVTGLSTTVDRTSSDAFRWATGNSTTSSDGRIFELPNYDEPVDQPQSAGRQRQSTVTVTREARVNNIFKIYSSTFDYQSIRDWVKGSYFVDIMCQIFAAEAHRLELRDLLHETANVVSSIYDLRYGIMTPSITCSADFGKLYFNPGIDDATTVVIHQVSDRNDGDSSGGNTSDNCDRIDYSNRNYGNSGERNTPDNGDRIDESDRINGNCREDTERNVSNRNDDKCREDGERNVCDNGDRTDDSNKEISNYREDCDRNVSNTGDRIDETNRNHGNCREDTEINSSNNSEENTSHSSDENVPNHIDRNNENPSEFRDINSSSYNDAIVDNSSVPNNSNRNTRNNSDYDEAIDTVDEAIDYREINNSRYDEKLVDNTS
